# Granzyme B PET Imaging Predicts Response to Immunotherapy in a Diet-Induced Obesity Model of Breast Cancer

**DOI:** 10.2967/jnumed.124.268938

**Published:** 2025-07

**Authors:** Shannon E. Lynch, Corinne Crawford, Addison L. Hunt, Luke L. Sligh, Yujun Zhang, Lyse A. Norian, Benjamin M. Larimer, Suzanne E. Lapi, Anna G. Sorace

**Affiliations:** 1 Graduate Biomedical Sciences, University of Alabama at Birmingham, Birmingham, Alabama;; 2 Department of Radiology, University of Alabama at Birmingham, Birmingham, Alabama;; 3 Department of Biomedical Engineering, University of Alabama at Birmingham, Birmingham, Alabama;; 4 O’Neal Comprehensive Cancer Center, University of Alabama at Birmingham, Birmingham, Alabama;; 5 Department of Pathology, University of Alabama at Birmingham, Birmingham, Alabama;; 6 Nutrition Obesity Research Center, University of Alabama at Birmingham, Birmingham, Alabama; and; 7 Department of Chemistry, University of Alabama at Birmingham, Birmingham, Alabama

**Keywords:** GZP PET imaging, TNBC, obesity, immunotherapy, granzyme B

## Abstract

The effects of obesity on cancer treatment efficacy remain unclear, as both beneficial and detrimental modulations of the tumor immune microenvironment have been reported. We compared ^68^Ga-NOTA-GZP (βAla–Gly–Gly–Ile–Glu–Phe–Asp–CHO) PET images with those obtained with the gold standard, ^18^F-FDG PET, to quantify biologic variations in a diet-induced obesity model of triple-negative breast cancer to understand how obesity influences the tumor immune landscape and response to immunotherapy. **Methods:** C57BL6/J mice were fed a high-fat diet (*n* = 24) or low-fat diet (*n* = 18) for 14 wk. EO771 tumor-bearing mice were treated with a fixed or weight-based dose of saline or checkpoint-blockade immunotherapy, and tumor volume was evaluated for long-term response. Mice were imaged via ^68^Ga-NOTA-GZP PET on day 7 to quantify immune activation, and those images were compared with ^18^F-FDG PET images to characterize changes in glucose metabolism on days 0 and 6. SUV was quantified from imaging data, and a cohort of mice was euthanized to validate biologic changes via flow cytometry. **Results:** Mice fed a high-fat diet demonstrated increased tumor glucose metabolism at baseline, as measured by ^18^F-FDG PET. No significant differences were observed in ^18^F-FDG SUV for responder tumors on day 6. The ^68^Ga-NOTA-GZP PET signal was increased in tumors responsive to immunotherapy on day 7 and was highly sensitive in predicting response via analysis of receiver-operating-characteristic curves. **Conclusion:** Obesity decreases response to immunotherapy by altering metabolism and the tumor immune microenvironment. ^68^Ga-NOTA-GZP PET imaging is a sensitive and predictive imaging biomarker of immunotherapy response, but weight-based dosing is needed to achieve effective changes in tumor volume.

## Visual Abstract

**Figure:**
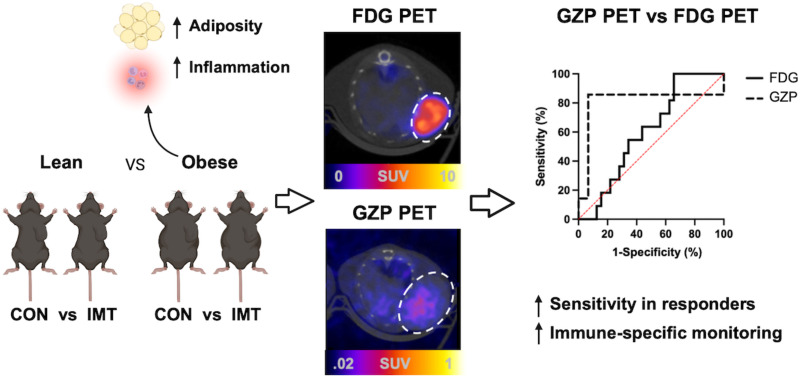

Cancer and obesity are fast-growing epidemics in the United States and globally (1). Breast cancer is the most prevalent cancer in women worldwide, with over 300,000 new diagnoses predicted for 2025 (2,3). Obesity is a risk factor associated with the development of postmenopausal breast cancer and increased disease progression and recurrence (4,5). Obesity worsens clinical outcomes and decreases overall survival for patients with triple-negative breast cancer (TNBC), with one study finding an 11% increased risk of mortality (6,7). Obesity is highly prevalent in the United States, affecting 41.9% of adults and 19.7% of adolescents as of 2020 (8,9). Obesity is a chronic inflammatory state known to promote tumor growth and exhaustive phenotypes, which greatly influences treatment response; however, the specific mechanisms contributing to an increased risk for developing cancer have yet to be elucidated. Moreover, obesity contributes to unique challenges in diagnosing, imaging, and treating patients with breast cancer (10,11). Specifically, it is not well understood how obesity affects immunotherapy response, and current studies aim to characterize this complex tumor biology and optimize response.

The current standard-of-care treatment for TNBC includes surgery, chemotherapy, radiation, and immunotherapy (12,13). Clinical dosing for checkpoint-blockade immunotherapy was initially weight-based but, as of 2017, is administered as a fixed dose to improve convenience and reduce waste while preserving efficacy (14–16). Some studies have demonstrated the potential benefits of weight-based dosing, including reduced financial burden, limited overexposure, off-target effects, and the personalization of medical treatments. Many trials and preclinical models have demonstrated the benefit of fixed-dose immunotherapy administration in non–small cell lung cancer, renal cell carcinoma, melanoma, urothelial cancer, head and neck cancer, and colorectal cancer, suggesting a reversal of immune dysfunction associated with inhibited and exhausted T-cell phenotypes seen in obesity (17–20). Contrastingly, studies have shown no differences in response or progression-free survival and have indicated that there are different pharmacokinetic properties for patients with obesity who receive fixed-dose immunotherapy, suggesting this regimen may not be optimal for these patients (14,21,22). The concept that obesity induces immune dysfunction but increases immunotherapy effectiveness is referred to as the “obesity paradox,” which was first described in 2018 by McQuade et al. (23,24). Due to the paradoxical nature of the response to immunotherapy, we investigated whether immunotherapy dosing influences tumor response in preclinical obesity models. Furthermore, we sought to identify imaging biomarkers that can be evaluated noninvasively and predict response to immunotherapy to enhance overall response.

Medical imaging is key to cancer diagnosis and treatment, but there are certain limitations and considerations for patients with obesity, including acquisition parameters, image quality, and dosing of exogenous imaging agents (25,26). An additional challenge lies in distinguishing the response to immunotherapy, which varies greatly from chemotherapy and radiation, as these therapies rely on the immune system to mediate tumor killing. Many studies have shown that responder tumors have an increased ^18^F-FDG signal, which may be attributed to pseudoprogression, hyperprogression, or inflammation due to the influx of immune cells (27,28). However, in response to more traditional therapies, a decreased ^18^F-FDG PET signal is associated with response, highlighting the need for novel methods to determine response to immunotherapy (29–31). New imaging techniques to quantify immunotherapy-related innate and adaptive immune activation and infiltration, including B cells, CD8 T cells, CD4 T cells, and natural killer cells, have gained interest (32–35). In concordance with targeting effector populations, advancements have also been made in evaluating effector molecules, such as granzyme B (GZB) (36). Although significant improvements have been made, the challenge of identifying biomarkers and predictors of response that are unique to immunotherapy remains. Consensus guideline criteria (iRECIST) have been adapted to objectively evaluate immunotherapy response and disease progression in solid tumors instead of strictly evaluating physical changes in tumor burden (37). However, not all patients respond well to immunotherapy, and therefore, identifying biomarkers that predict treatment response could help distinguish patients who may be good candidates for this therapy and greatly improve the effectiveness of immunotherapy.

The goal of our study was to use molecular imaging of immune changes to characterize obesity-induced differences in the breast cancer microenvironment at baseline and throughout the course of immunotherapy. We also explored the effects of different dosing strategies on tumor growth kinetics and the immune landscape in a diet-induced obesity model of TNBC. We compared differences in immune activation and glucose metabolism, which are key mediators of tumor growth and response to therapy. Through the use of noninvasive imaging, we aimed to characterize underlying cellular and molecular differences induced by obesity that influence treatment decisions and have the potential to optimize and predict the response to checkpoint-blockade immunotherapy.

## MATERIALS AND METHODS

### Cell Culture

EO771 mammary carcinoma cells were purchased from ATCC (CRL-3461) and confirmed to be estrogen receptor negative, progesterone receptor negative, and human epidermal growth factor receptor 2 negative via Western blot (Supplemental Fig. 1) (supplemental materials are available at http://jnm.snmjournals.org).

### Animal Models

All procedures involving mice were approved by the University of Alabama at Birmingham’s institutional animal care and use committee (APN 08778). Forty-one female C57BL/6J mice (age, 5–6 wk) were randomized to receive either a high-fat diet (HFD; 60% kcal from fat) (RD12492i; Research Diets) (*n* = 24) or low-fat diet (LFD; 10% kcal from fat) (RD12450Ji; Research Diets) (*n* = 17) for 14 wk to induce obesity (Supplemental Fig. 2). Body weight and food intake were measured once weekly, and the diets (150 g/wk) were administered to the entire cage. Mice were housed 5 per cage in a standard 12-h light–dark cycle. We implanted 5 × 10^5^ EO771 cells in 20% Matrigel matrix (Corning) into the third mammary fat pad of all mice roughly 2 wk before imaging studies. Additional expanded details are provided in the supplemental materials.

### Treatments

Mice were randomized into treatment groups to achieve an average group volume of 150 mm^3^. Checkpoint-blockade immunotherapy consisted of anti-cytotoxic T-lymphocyte 4 (BE0131; BioXCell) and anti-programmed death 1 (BE0146; BioXCell) and were administered every 3 d via intraperitoneal injection. Groups received 1 of 5 treatments: LFD plus saline control (*n* = 7); LFD plus fixed-dose immunotherapy, consisting of 100 µg of anti-cytotoxic T-lymphocyte 4 and 200 µg of anti-programmed death 1 (*n* = 10); HFD plus saline control (*n* = 7); HFD plus fixed-dose immunotherapy (*n* = 8); or HFD plus weight-based equivalent of fixed-dose immunotherapy, consisting of 200 µg of anti-cytotoxic T-lymphocyte 4 and 400 µg of anti-programmed death 1 (*n* = 9). A threshold of response was calculated based on the average tumor volume of the saline control group plus 1 SD, and any tumor volume below that threshold was deemed a responder tumor. Due to variable immunotherapy response, tumors were further categorized on the basis of iRECIST criteria with caliper measurements, as previously described (37).

### Biologic Validation

A cohort of mice (*n* = 3 or 4 per group) was euthanized on day 7 for biologic validation studies. Tumors were digested into single-cell suspension and stained using fluorophore-labeled antibodies to probe immune populations via flow cytometry. Supplemental Table 1 lists the antibodies and dilutions used. Flow cytometry analysis parameters, including fluorescence minus one controls used for gating, are shown in Supplemental Figure 3. Supernatants were collected from tumor tissue during digestion for further cytokine analysis. Additional details are provided in the supplemental materials.

### Radiotracer Synthesis, Image Acquisition, and Analysis

^18^F-FDG was purchased from Cardinal Health, a commercially available source for radiotherapeutics. A probe for targeting GZB peptide, NOTA-GZP (NOTA–βAla–Gly–Gly–Ile–Glu–Phe–Asp–CHO), was labeled with ^68^Ga eluted from a commercially available ^68^Ge/^68^Ga generator (Eckert & Ziegler). Radiolabeling efficiency was determined using instant thin-layer chromatography, and radiochemical yields with a purity of greater than 95% were used for in vivo studies.

Mice were intravenously injected with 3.7 MBq (100 μCi) of ^18^F-FDG PET on days 0 and 6 and 5.5 MBq (150 μCi/1 µg) of ^68^Ga-NOTA-GZP PET on day 7 of immunotherapy treatment (Supplemental Fig. 2). A 20-min PET image acquisition, followed by a 5-min CT scan for anatomic reference, was performed on a small-animal PET/CT (SOFIE). SUVs were quantified (VivoQuant version 4.0), and histograms of SUV voxelwise data were used to calculate the percentage of GZP plus effector cell fraction (positive threshold identified by muscle plus 1 SD). Additional details about image analysis are provided in the supplemental materials.

### Statistical Analysis

All analyses were performed in GraphPad Prism, version 10. One-way independent ANOVAs were used to compare differences across treatment groups, and nonparametric *t* tests were used to assess differences between diets.

## RESULTS

### Obesity-Induced Changes in Tumor Microenvironment

**FIGURE 1.**
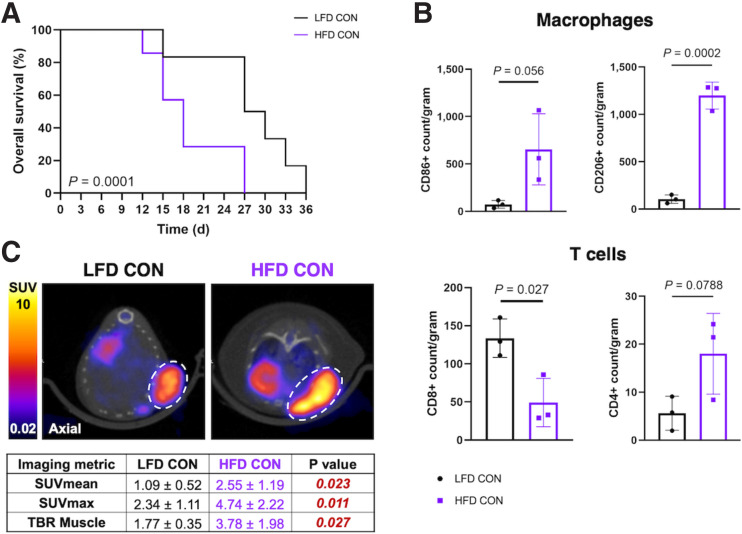
Obesity induces changes in tumor microenvironment that influence survival. (A) Overall survival was significantly decreased for HFD-fed mice (median, 18 d) compared with LFD-fed mice (median, 28.5 d). (B) Quantification of flow cytometry data evaluating macrophage (CD86^+^ and CD206^+^) and T-cell (CD4^+^ and CD8^+^) populations in tumors of lean and obese mice. Macrophages are gated on live > CD45^+^ > CD11b^+^ > F4/80^+^, and T cells are gated on live > CD45^+^ > CD3^+^ (Supplemental Fig. 3). (C) ^18^F-FDG PET/CT imaging and quantification of SUV metrics show tumor glucose metabolism in lean and obese mice at baseline. Tumors are encircled with white dotted line. CON = control; TBR = tumor background ratio.

**FIGURE 2.**
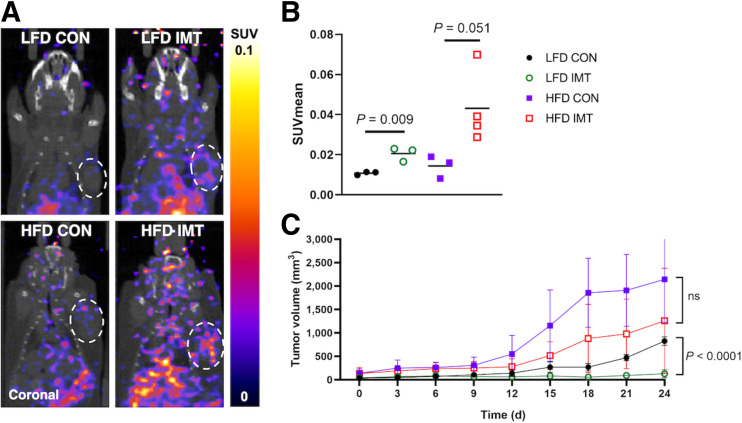
Fixed-dose immunotherapy significantly increased immune activation via GZB PET but was not sufficient to reduce tumor burden in obese mice. (A) Representative PET/CT images show coronal view of EO771 mice tumors imaged with ^68^Ga-NOTA-GZP PET. (B) ^68^Ga-NOTA-GZP SUV_mean_ in tumors treated with fixed-dose immunotherapy compared with controls for LFD and HFD groups. (C) Tumor volume reduction by fixed-dose immunotherapy observed in LFD-fed mice but not HFD-fed mice. CON = control; IMT = immunotherapy.

HFD-fed mice had significantly increased body weight (*P* < 0.0001; Supplemental Fig. 4A), and tumor-bearing mice fed the HFD had reduced survival compared with their lean counterparts (*P* = 0.0001; Fig. 1A). Quantification of macrophage and T**-**cell populations (Fig. 2B) shows significant differences between LFD-fed and HFD-fed tumor microenvironments, which play a key role in mediating tumor response to immunotherapy. HFD-fed mice had significantly increased CD86^+^ (*P* = 0.05) and CD206^+^ (*P* = 0.0002) macrophages per gram of tumor compared with their lean counterparts (Fig. 1B). CD86^+^ M1-like macrophages typically have a proinflammatory phenotype, whereas CD206^+^ M2-like macrophages are antiinflammatory and associated with tumor progression by promoting angiogenesis and extracellular matrix remodeling (38,39). Compared with their lean counterparts, HFD-fed mice had increased CD4^+^ T cells, which trended toward significance (*P* = 0.07) and significantly decreased CD8^+^ T cells (*P* = 0.02) (Fig. 1B). CD8^+^ T cells are well known to be antitumoral and have cytotoxic effector capabilities, whereas the properties of CD4^+^ T**-**helper cells vary based on subtype (40,41). Flow cytometry data indicated that obesity promoted inflammatory, protumoral immune populations, which may have contributed to treatment response. Tumor glucose metabolism, as measured via ^18^F-FDG PET, was significantly elevated at baseline for HFD-fed mice compared with LFD-fed mice (*P* = 0.02; Fig. 1C). ^18^F-FDG PET showed increased average glucose metabolism (SUV_mean_) in tumors of obese mice compared with their lean counterparts at baseline (*P* = 0.0001; Fig. 1C). Taken together, imaging and flow cytometry data suggested that obesity induced physical and metabolic differences in tumors, specifically by increasing glucose metabolism and promoting protumoral and inflammatory innate and adaptive immune populations.

### Fixed-Dose Immunotherapy

PET imaging with ^68^Ga-NOTA-GZP after fixed-dose immunotherapy showed that effector-cell activation via GZB was significantly increased for both LFD-fed and HFD-fed mice (Fig. 2A). Quantification of ^68^Ga-NOTA-GZP uptake showed that tumor SUV_mean_ was significantly increased for LFD-fed (*P* = 0.009) and HFD-fed (*P* = 0.05) mice treated with immunotherapy compared with those who received the saline control (Fig. 2B). However, fixed-dose immunotherapy did not effectively reduce tumor burden longitudinally for HFD-fed mice (*P* = 0.19) but did result in significant reductions in in LFD-fed mice (*P* < 0.0001) (Fig. 2C). Fixed-dose immunotherapy increased immune activation but did not reduce tumor burden in obese mice.

### Weight-Based Immunotherapy

**FIGURE 3.**
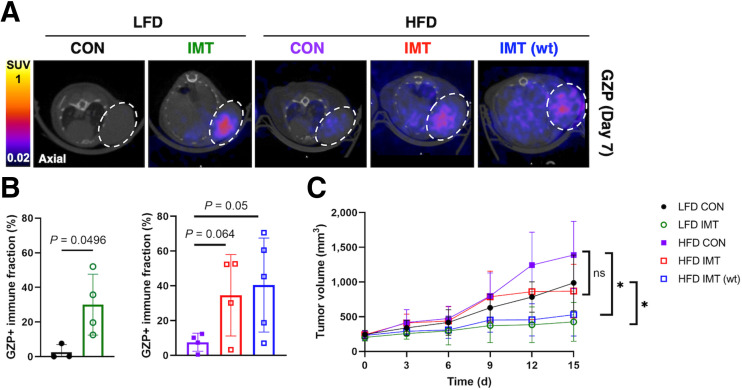
Weight-based immunotherapy significantly increased immune activation via ^68^Ga-NOTA-GZP PET. (A) PET/CT images showing axial view of EO771 mice tumors imaged with ^68^Ga-NOTA-GZP PET. (B) Frequency distribution for ^68^Ga-NOTA-GZP expression. Percent GZP^+^ effector cell fraction calculated from SUV frequency distribution for [^68^Ga]Ga-NOTA-GZP expression in lean and obese mice. (C) Tumor volumes were significantly decreased for mice receiving weight-based immunotherapy compared with controls. CON = control; IMT = immunotherapy; wt = weight.

Quantitative biologic validation of imaging data via flow cytometry demonstrated that weight-based dosing most effectively increased CD8^+^ T-cell (Supplemental Fig. 5A) and CD8^+^ GZB^+^ effector populations (Supplemental Fig. 5B) and influenced CD4^+^ T-cell populations (Supplemental Fig. 5C) in obese mice (Supplemental Figs. 5A–5C). Cytokine analysis of tumor supernatant revealed that immunotherapy also significantly increased T-helper type 1 antitumor subsets and decreased T-helper type 2 protumor subsets of CD4 T cells to mediate response to immunotherapy (Supplemental Figs. 5D and 5E). ^68^Ga-NOTA-GZP PET showed that both fixed-dose and weight-based dosing increased ^68^Ga-NOTA-GZP uptake in the tumors of obese mice (Fig. 3A). Voxelwise analysis of the frequency distributions of GZP uptake showed that immunotherapy altered the distribution of ^68^Ga-NOTA-GZP^+^ voxels in lean and obese mice compared with control mice (Supplemental Figs. 6A–6C). Quantification of the percentage of positive voxels from histogram distributions of GZP uptake demonstrated that fixed-dose immunotherapy significantly increased the GZP^+^ effector cell fraction in the tumors of lean (*P* = 0.04) and obese (*P* = 0.05) mice (Fig. 3B). In addition to increasing immune activation via ^68^Ga-NOTA-GZP, weight-based immunotherapy significantly reduced tumor volume compared with mice in the control group who were fed the HFD (*P* = 0.007) and LFD (*P* = 0.002) (Fig. 3C). Mice receiving fixed-dose immunotherapy had no significant difference in tumor volume compared with control mice (*P* = 0.12; Fig. 3C). Weight-based immunotherapy dosing more significantly altered GZB activation and decreased tumor volume compared with fixed-dosing in obese mice without inducing toxicity. Body weight, as a measure of toxicity, did not significantly differ between mice receiving weight-based dosing of immunotherapy compared and those receiving a fixed-dose or those in the control groups (Supplemental Figs. 4B and 4C; *P* = 0.41).

### ^68^Ga-NOTA-GZP PET and Response to Immunotherapy

**FIGURE 4.**
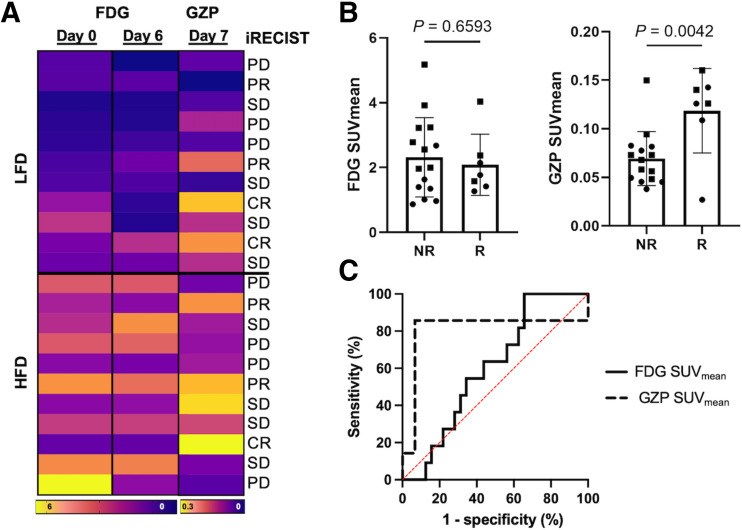
^68^Ga-NOTA-GZP PET was significantly associated with response to immunotherapy before significant changes in tumor volume. (A) Heat map displaying ^18^F-FDG SUV on days 0 and 6, ^68^Ga-NOTA-GZP SUV on day 7, and relationship with iRECIST criteria for each mouse. (B) Comparison of responder and nonresponder tumors with day 6 ^18^F-FDG SUV_mean_ and day 7 ^68^Ga-NOTA-GZP SUV_mean_. (C) Receiver-operating-characteristic curve analysis shows sensitivity and specificity in predicting response of ^18^F-FDG SUV_mean_ (area under the curve, 0.6023; 95% CI, 0.5472–1.000; *P* = 0.31) and ^68^Ga-NOTA-GZP SUV_mean_ (area under the curve, 0.8095; 95% CI, 0.4311–0.7344; *P* = 0.022). CR = complete response; NR = nonresponder; PD = progressive disease; PR = partial response; R = responder; SD = stable disease.

Responder and nonresponder mice were identified using iRECIST criteria and evaluated for differences in imaging metrics to determine predictors of response (Fig. 4A). ^18^F-FDG PET SUV_mean_ on day 6 of treatment was not significantly different between responders and nonresponders (*P* = 0.66; Fig. 4B). The ^68^Ga-NOTA-GZP PET SUV_mean_ obtained on day 7 of treatment was significantly increased in responder tumors compared with nonresponders treated with immunotherapy (*P* = 0.0042; Fig. 4B). Receiver-operating-characteristic curve analysis showed sensitivity and specificity of ^18^F-FDG and ^68^Ga-NOTA-GZP SUVs in predicting response to therapy. The area under the curve was 0.623 for ^18^F-FDG and 0.8095 for ^68^Ga-NOTA-GZP (Fig. 4C). The ^68^Ga-NOTA-GZP PET signal was highly sensitive in determining response to immunotherapy before tumor volume changes, shown via receiver-operating-characteristic curve analysis (*P* = 0.02).

### Change in ^18^F-FDG PET Signal in Responder Tumors

**FIGURE 5.**
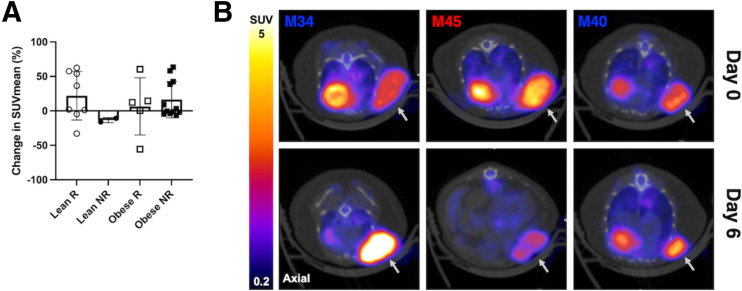
Responder tumors showed high variance in change in ^18^F-FDG signal during treatment in lean and obese mice. (A) Percent change in ^18^F-FDG SUV_mean_ from day 0 to day 6 for responder (R) and nonresponder (NR) tumors. (B) ^18^F-FDG PET/CT images in axial view of responder tumors in obese mice on days 0 and 6. Gray arrows indicate tumors. M34, M45, and M40 = mouse number; NR = nonresponder; R = responder.

^18^F-FDG SUV_mean_ was not significantly different in responder tumors compared with nonresponder tumors in lean or obese mice; however, responder tumors showed a high variability in change in ^18^F-FDG signal from day 0 to day 6 (Fig. 5A). The range of percent change in ^18^F-FDG SUV_mean_ for lean mice was 93.47 for responders and 5.24 for nonresponders. The range of percent change in ^18^F-FDG SUV_mean_ for obese mice was 116.1 for responders and 70.26 for nonresponders (Fig. 5A). The ^18^F-FDG SUV_mean_ increased in some responder tumors but decreased in others (Fig. 5B). Similar to other studies (42–44), we observed a high variance of change in ^18^F-FDG signal in response to immunotherapy, with even greater variance observed in obese mice.

## DISCUSSION

Obesity is a chronic inflammatory state characterized by high levels of adipose tissue, which is associated with dysregulated hormone and appetite signaling through leptin and ghrelin (39,45). Obesity has been associated with increased secretion of inflammatory cytokines and dysfunctional adaptive immune functions (38,46). Our studies support current findings which suggest that obesity creates an inflammatory, protumoral shift in the innate and adaptive immune populations within the tumor microenvironment (4,24,40). Most notably, we observed a significant increase in CD86^+^ and CD206^+^ macrophages and CD4^+^ T cells, with decreased CD8^+^ T cells in tumors of obese mice when compared with their lean counterparts. In addition to baseline alterations in the tumor immune landscape, obesity greatly influences the efficacy of checkpoint-blockade immunotherapy, which largely relies on effector immune populations (e.g., CD8^+^ T cells). Compared with LFD-fed mice, HFD-fed mice in both immunotherapy-treated groups had significantly decreased CD8^+^ tumor-infiltrating lymphocytes and GZB^+^ CD8^+^ effector T cells. However, quantification of GZP^+^ effector cell fraction from PET imaging showed that the ^68^Ga-NOTA-GZP signal had significantly increased in all immunotherapy-treated groups in both LFD- and HFD-fed mice. Increased ^68^Ga-NOTA-GZP signal without an increase in CD8^+^ GZB^+^ cells in the HFD cohort suggests that other effector immune populations, such as natural killer cells, may be responsible for secreting GZB. This highlights the inflammatory nature of the tumor immune microenvironment in obese mice compared with their lean counterparts, which may contribute to poor outcomes. Importantly, we observed that early imaging (on day 7) with ^68^Ga-NOTA-GZP PET during immunotherapy was highly sensitive for predicting tumor response long-term. Although obesity significantly increased ^18^F-FDG uptake at baseline, ^18^F-FDG was not an effective biomarker for distinguishing responder tumors from nonresponder tumors. Increased ^18^F-FDG signal in the tumors of obese mice can be indicative of alterations in glucose metabolism but may also be attributed to increased or chronic states of inflammation induced by diet alterations. ^18^F-FDG is nonspecific and is often associated with an influx of immune cells that metabolize glucose, congruent with our flow cytometry data showing increased populations of tumor-promoting lymphocytes.

^18^F-FDG PET, the gold standard for diagnosis and tumor monitoring, is known to have variable changes due to many factors such as inflammation and immune infiltration (42,43,47). Interestingly, ^18^F-FDG uptake after immunotherapy and the change in ^18^F-FDG signal from day 0 to day 6 did not significantly differ between responder and nonresponder tumors. A recent study noted that the change in ^18^F-FDG signal between 2 consecutive PET scans decreased because of metabolic response and increased in the setting of metabolic flare and was predictive of response to immunotherapy in melanoma (44). That study was highly impactful as it suggested that metabolic changes, rather than simply decreases in ^18^F-FDG signal, are significantly correlated with progression-free survival. Additional analysis in our model showed similar variability in ^18^F-FDG signal changes from day 0 to day 6 in responder tumors in both lean and obese mice. Our PET imaging data indicate that obesity increases tumor glucose metabolism at baseline, which may have a considerable effect on the ^18^F-FDG PET signal as a determinant of response.

The ^68^Ga-NOTA-GZP PET signal was significantly increased for obese mice with responder tumors and correlated with end tumor volume, which demonstrates that ^68^Ga-NOTA-GZP is a robust biomarker, as it has been previously predictive of response in lean mice (41). The ^68^Ga-NOTA-GZP PET signal was strongly correlated with CD8^+^ GZB^+^ T cells in flow cytometry, highlighting the accuracy of ^68^Ga-NOTA-GZP in evaluating effector cell activation noninvasively. The ^68^Ga-NOTA-GZP PET signal showed significant distinctions in responder and nonresponder tumors early in the course of immunotherapy treatment and before changes in tumor volume, revealing ^68^Ga-NOTA-GZP to be a sensitive biomarker of immunotherapy response, even in a state of chronic inflammation. ^68^Ga-NOTA-GZP PET may have potential as a predictive imaging biomarker for immunotherapy response in other models or chronic inflammatory conditions. Our studies also indicate that weight-based administration of immunotherapy may be sufficient to overcome the inflammatory tumor immune landscape induced by obesity. Fixed-dose immunotherapy did not reduce tumor burden for obese mice, but weight-based immunotherapy administration significantly reduced tumor burden for HFD-fed mice while maintaining increased immune activation via GZB. Specifically, weight-based dosing more effectively stimulated the secretion of GZB serine proteases compared with fixed-dose regimens in obese models. Importantly, this difference in immune activation was determined noninvasively via ^68^Ga-NOTA-GZP PET, which could inform response to immunotherapy and improve patient stratification for response and reduce unnecessary, ineffective treatment administration clinically (48,49). Determining the response to immunotherapy is difficult, and identifying imaging biomarkers that predict response is a clinically relevant approach to personalize treatment and improve response.

A limitation of this study lies in the examination of potential toxicity induced by weight-based administration of immunotherapy. For patients with TNBC, anti-programmed death 1 immunotherapy is administered as a flat dose of 200 mg every 3 wk or 400 mg every 6 wk, similar to our fixed-dose regimen (50). Toxicity and off-target effects, ranging from common side effects (e.g., pneumonitis, colitis, diarrhea, hepatitis, dermatologic effects) to rare toxicities (e.g., nephrotoxicity, cardiotoxicity, neurologic effects, death) are major concerns for patients receiving immunotherapy (51). Although we did not perform tissue-specific toxicity assessments, we used weight and body condition scores as determinants of overall health for the mice studied. We observed no significant changes in body weight or body condition among mice receiving weight-based or fixed-dose immunotherapy or the saline control. Studies to assess potential toxicity of this therapy in diet-induced obesity models will be important for future assessment. The original dosing recommendations for immunotherapy were weight-based, but the current standard for administration is a fixed-dose regimen. Recommended dosing was shifted to improve convenience and reduce resource waste while preserving treatment efficacy (14,15,19). However, our study has indicated that escalating the dose of immunotherapy improves outcomes preclinically and may be beneficial for patients with obesity. Influencing clinical care will be difficult, as the literature has not shown any indication of negative implications for patients with obesity receiving fixed-dose immunotherapy.

## CONCLUSION

Obesity contributes to resistance to immunotherapy in preclinical models of TNBC by increasing tumor glucose metabolism and altering the tumor immune landscape. Obesity promotes inflammatory, protumoral populations, including CD206^+^ macrophages and CD4^+^ T cells. Although responses were variable, weight-based dosing of immunotherapy significantly altered immune activation via GZB, which can be quantified noninvasively via ^68^Ga-NOTA-GZP PET. ^68^Ga-NOTA-GZP PET revealed differences in immune activation in obese and lean mice and during dosing of immunotherapy in obese mice. Importantly, the ^68^Ga-NOTA-GZP PET signal was predictive of response to immunotherapy before significant changes were seen in tumor volume in a chronic inflammatory state. Understanding complex tumor immune biology at baseline and throughout therapy may inform imaging-directed treatment decision-making.

## DISCLOSURE

Financial support for this work was provided by the National Institutes of Health National Cancer Institute (R01CA240589, R01CA276540, R01CA279143) and the University of Alabama at Birmingham O’Neal Comprehensive Cancer Center Preclinical Imaging Shared Facility Grant (P30CA013148). The content is solely the responsibility of the authors and does not necessarily represent the official views of the NIH. Benjamin Larimer is the cofounder and CEO of CytoSite Biopharma. No other potential conflict of interest relevant to this article was reported.

## Notes

KEY POINTS **QUESTION:** Can ^68^Ga-NOTA-GZP PET imaging predict response to immunotherapy in a diet-induced obesity model of TNBC? **PERTINENT FINDINGS:** In a diet-induced obesity model of EO771 tumor-bearing mice, obese mice revealed decreased response to immunotherapy and an increased inflammatory, protumor immune landscape compared with their lean counterparts. Weight-based dosing of immunotherapy enhanced effector cell activation, as measured by the ^68^Ga-NOTA-GZP PET signal, which correlated to decreased tumor burden longitudinally. **IMPLICATIONS FOR PATIENT CARE:** Although obesity dampens response to immunotherapy by altering metabolism and the tumor immune microenvironment, ^68^Ga-NOTA-GZP PET imaging serves as a sensitive, predictive imaging biomarker of response.
